# A New Statistical Framework for Corpus Callosum Sub-Region Characterization Based on LBP Texture in Patients With Parkinsonian Disorders: A Pilot Study

**DOI:** 10.3389/fnins.2020.00477

**Published:** 2020-05-28

**Authors:** Debanjali Bhattacharya, Neelam Sinha, Shweta Prasad, Pramod Kumar Pal, Jitender Saini, Sandhya Mangalore

**Affiliations:** ^1^Department of Networking and Communication, International Institute of Information Technology, Bangalore, India; ^2^Department of Neurology, National Institute of Mental Health and Neuroscience, Bangalore, India; ^3^Department of Clinical Neurosciences, National Institute of Mental Health and Neuroscience, Bangalore, India; ^4^Department of Neuroimaging and Interventional Radiology, National Institute of Mental Health and Neuroscience, Bangalore, India

**Keywords:** Parkinsonian disorders, corpus callosum, local binary pattern, support vector machine, scatter index

## Abstract

**Purpose:**

The study is conducted to identify the best corpus callosum (CC) sub-region that corresponds to highest callosal tissue alteration occurred due to Parkinsonism. In this regard the efficacy of local binary pattern (LBP) based texture analysis (TA) of CC is performed to quantify the changes in topographical distribution of callosal fiber connected to different regions of cortex. The extent of highest texture alteration in CC is used for differential diagnosis.

**Materials and Methods:**

Study included subjects with Parkinson’s disease (PD) (*n* = 20), and atypical Parkinsonian disorders – multiple system atrophy (MSA) (*n* = 20), Progressive supranuclear palsy (PSP) (*n* = 20), and healthy controls (*n* = 20). For each subject, we have automated the ROI extraction within mid-sagittal CC, followed by LBP TA. Two-class support vector machine (SVM) classification for each disorder as against HC is performed using extracted LBP features like energy and entropy. Correct classification ratio (CCR) is computed as the fraction of correctly classified ROIs at each of the CC sub-regions based on well-known Witelson and Hofer schemes. Based on CCR values, the “Scatter Index (SI)” is proposed to capture how localized (closer to 0) or scattered (closer to 1) the textural changes are among the CC sub-regions, across all subjects per class. The CCR values are further utilized to classify the disease groups.

**Results:**

Highest alteration of texture is observed in mid-body of CC. The consistency of this finding is quantified using SI for all subjects in a specific class that results more localized textural changes in PSP (15%) and MSA (25%), in comparison to PD (47%). Classification among disease groups results maximum classification accuracy of 90% in classifying PSP from PD-NC.

**Conclusion:**

Our result demonstrates the efficacy of proposed methodology in analyzing tissue alteration in MRI of Parkinsonian disorders and thus has potential to become valuable tool in computer aided differential diagnosis.

## Introduction

Parkinsonian disorders are chronic progressive neurodegenerative movement disorders, which are classically categorized into Parkinson’s disease (PD) and atypical Parkinsonian disorders such as Progressive supranuclear palsy (PSP), multiple system atrophy (MSA), etc. (Williams and Litvan, 2013). PD is typically characterized by levodopa responsive rigidity, bradykinesia, and tremor, whereas atypical Parkinsonian disorders present with several other motor systems and tend to be poorly responsive to levodopa. These disorders have been extensively studied using a multitude of structural neuroimaging sequences and modalities of analysis (Paviour et al., 2006; Heim et al., 2018; Rispoli et al., 2018); however, texture analysis (TA), which is a quantitative method of characterizing tissue types based on texture, has seldom been performed for Parkinsonian disorders.

In image processing, TA refers to the characterization of regions in an image by their texture content. The term “texture” can be defined as a set of primitive texels (texture elements) arranged in a particular spatial relationship. Thus TA aims to derive an effective quantitative description of textures by extracting various texture features. For analyzing medical images TA is highly significant especially when tissue alteration cannot be visually perceived, making it a powerful tool for computer aided medical image diagnosis. Several studies have reported the diagnostic utility of TA for different types of neurological diseases like Alzheimer’s disease and other Dementia (Freeborough and Fox, 1998; Kodama et al., 2009; Sivapriya et al., 2011). In diseases such as non-Hodgkin lymphoma, mild traumatic brain injury, and multiple sclerosis, TA has been able to identify lesions which are not easily identifiable by the naked eye (Harrison, 2011).

The corpus callosum (CC) is the principal white matter fiber tract which connects the two cerebral hemispheres and is relatively resistant to age-related changes in healthy individuals (Bishop and Wahlsten, 1997). The integrity of callosal fibers is associated with the communication of nerve signals between the two cerebral hemispheres. This communication can be potentially disrupted due to neurodegeneration leading to cognitive impairments. Studies on CC morphometry (Luders et al., 2018), volumetric and diffusion abnormalities of the CC have been previously reported in Parkinsonian disorders (Lenka et al., 2017). Furthering these advancements, our study characterizes CC sub-regions based on its texture content.

**FIGURE 1:**
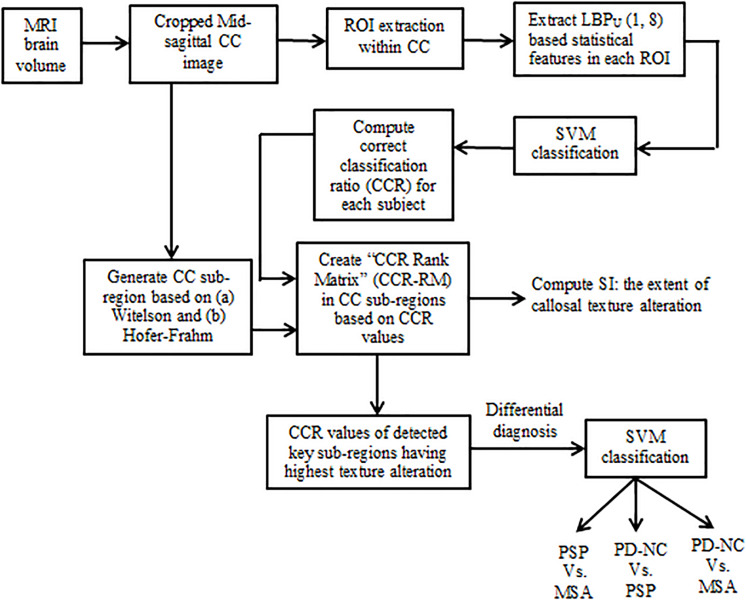
Block schematic of our proposed methodology.

The primary aim of the current study is to isolate that CC sub-region which manifests to highest callosal tissue alteration occurred due to Parkinsonism. In this regard we perform local binary pattern (LBP) based TA of the CC in patients with Parkinsonian disorders. This enlists information about the integrity of the CC. Subsequently in order to check the consistency of findings across all subjects, we derive a novel statistical framework. This framework answers “*how localized or scattered the maximum tissue alteration is within CC*” across all subjects belonging to a specific class. The results show that significant differences exist between Parkinsonian disorders. This can be exploited to develop potential tools for differential diagnosis of Parkinsonian disorders. The block schematic of the proposed methodology is shown in Figure 1.

## Materials and Methods

### Subject Recruitment and Clinical Evaluation

**FIGURE 2:**
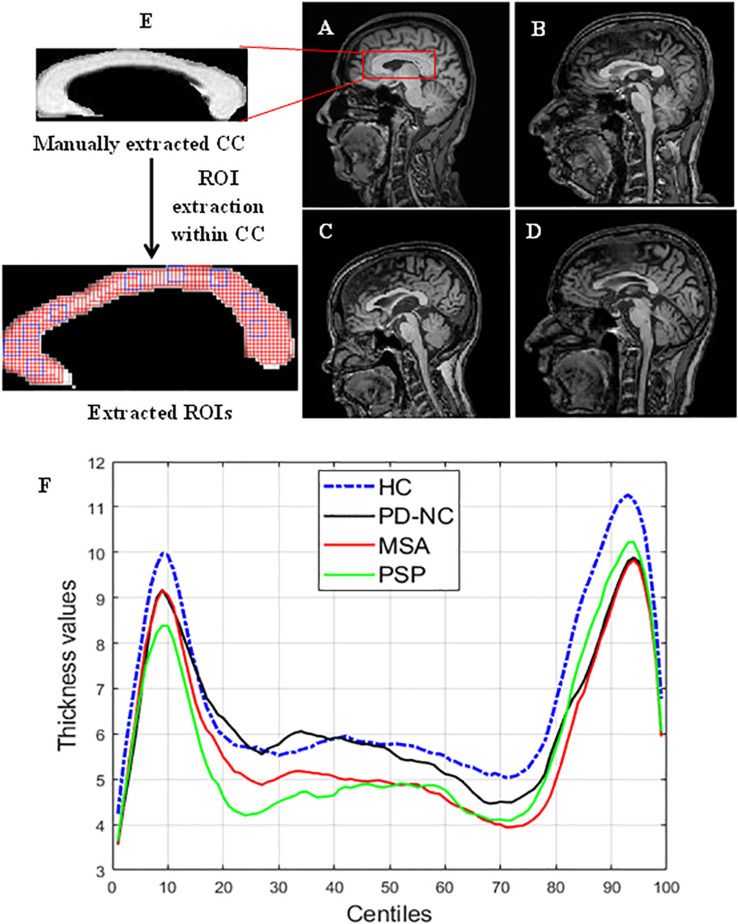
Comparison of CC region in brain across samples taken from HC. PD, PSP, and MSA **(A)** MR image of HC. **(B–D)** MRI scans of subjects with PD. PSP and MSA respectively. Panel **(F)** shows the CC thickness centile profile for all study groups: HC (blue)-blue, PD-NC (black). MSA (red), PSP (green). The centiles are plotted along *x*-axis. The thickness profile clearly shows the thickness reduction in CC occurred in Parkinsonian disorders compared to HC. The zoomed version of (i) manually extracted CC from mid-sagittal MRI and (ii) the extracted ROIs within CC are also shown in the left most column. ROI (the overlapping rectangular boxes of size 5 × 5 shown in red all through the CC) automation with window size 5 × 5 ensures the entire CC is exhaustively covered. Some sample ROIs are highlighted in blue.

Twenty patients with cognitively normal PD (PD-NC), 20 patients with clinically probable PSP, 20 patients with probable MSA, and 20 healthy controls were recruited from the general outpatient clinic and movement disorder services at the Department of Neurology. The diagnosis of PD-NC was based on the UK Parkinson’s Disease Society Brain Bank criteria (Hughes et al., 1993), the diagnosis of MSA was based on the criteria by Gilman et al. (2008), and PSP was diagnosed based on the National Institute of Neurological Disorders and Stroke and Society for PSP criteria (Litvan et al., 1996), and confirmed by a trained movement disorder specialist (author PKP). Age and gender matched HCs with no family history of Parkinsonism or other movement disorder were recruited. Mid-sagittal MR images of brain for one subject of each class are shown in Figure 2. The study is approved by local ethics committee of the National Institute of Mental Health and Neuro Science. The demographic and clinical details of study groups are tabulated in Table 1.

**TABLE 1:** Demographic details of patients and controls.

	**PD-NC (*n* = 20)**	**MSA (*n* = 20)**	**PSP (*n* = 20)**	**HC (*n* = 20)**
Male: female	13:7	12:8	16:4	15:5
Mean age (in years)	59.15 ± 8.15	53.90 ± 5.53	63.50 ± 7.36	55.3 ± 2
Mean age at onset	52.07 ± 8.24	51.05 ± 5.60	61.32 ± 7.10	NA
Duration of illness	7.57 ± 4.30	2.85 ± 1.50	2.17 ± 0.79	NA
Mini Mental State Examination score (MMSE)	28.71 ± 0.84	27.52 ± 1.20	26.20 ± 2.30	NA

### Imaging

MR images were acquired on 3T Philips Achieva scanner with a 32-channel head coil at NIMHANS, Bangalore, India. T1 weighted Magnetization Prepared Rapid Gradient Echo (MPRAGE) sequence covering the whole brain with TR = 8.1 ms; TE = 3.7 ms; flip angle = 8°; sense factor = 3.5; field of view (FOV) = 256 mm × 256 mm × 155 mm; voxel size = 1 mm × 1 mm × 1 mm; slice thickness = 1 mm; acquisition matrix = 256 × 256.

### Extraction of Region of Interests Within CC

The entire CC is manually cropped from mid-sagittal MR images of brain for each subject. This is done using MATLAB (MathWorks), which is multi-paradigm numerical computing software in C++, java programming environment. A region of interest (ROI) is defined as a sub-image patch of CC that can capture the local callosal texture information. Hence in order to get information about the texture of entire CC, we have extracted all possible ROIs within CC. The execution of ROI extraction is automated for all subjects. ROIs are extracted carefully so that it does not include any pixels outside the boundary of the CC structure. The size of these ROIs is fixed and limited to 5 × 5 pixels in case of HC shown in Figure 2. It was empirically observed that ROIs larger than 5 × 5 could not cover the entire CC, without spilling out. Likewise for three disease groups the size of ROIs is fixed to 4 × 4 pixels due to thickness reduction that occurred in disease group. Changes in callosal mean thickness in Parkinsonian disorder with respect to control group are shown in Figure 2 where callosal thickness is measured at 100 points spaced by equal angles from a specified centroid located half way between the most anterior and posterior extents of the callosum and along the inferior–superior axis at the most inferior extent of the splenium. The mean thickness profile of CC for all groups is plotted using C8 toolbox (Timothy et al., 2012).

### TA Using LBP

TA using LBP is first mentioned by Harwood et al. (1993), and introduced by Ojala (1996). LBP (Ojala et al., 2001) is a widely used TA method that has been found very efficient in discriminating textures of MR images (Oppedal et al., 2015). The evidence obtained from the literature suggests that the local texture information obtained from LBP-based TA can be utilized to detect disease related abnormalities that may not be perceptually visible. This makes LBP a powerful tool in computer aided diagnosis for patients suffering from Parkinsonian disorders. The explanation of LBP-based TA is described in section “LBP Methodology.”

#### LBP Methodology

Local binary pattern operator encodes a local texture pattern from an image patch. Here, the texture “T” is defined as joint distribution of “P” number of gray levels of local neighborhood around an image pixel: *T* = *t*(*g_c_*,*g*_0_,…,*g_P−1_*) where *g_c_* denote the gray level of an arbitrary pixel (*x*, *y*) of image I, i.e., *g_c_* = I (*x*, *y*) and *g_P _*denote the gray value of a sampling point in an evenly spaced circular neighborhood of P sampling points and radius R around point (*x*, *y*) and is calculated as *g_P_* = *I*(*x_P_*,*y_P_*) where *x_P_* = *x* + *Rcos*(2π*p/P*) and *y_P_* = *y*−*Rsin*(2π*p/P*) for *p* = 0 to P-1. The function “t” eventually defined the texture of the image. In order to obtain a robust texture classification, LBP uses a local circular window. In a circular neighborhood, the intensity values of diagonal pixels, having non-integer pixel coordinates, are estimated by bilinear interpolation.

To achieve gray level invariance, the center pixel value is subtracted from all gray values of the circular neighborhood: *T* = *t*(*g*_*c*_,*g*_0_,…,*g*_*P*−1_-*g*_*c*_) Assuming the center pixel to be statistically independent of the differences, we can factorize the above as: *T*≈*t*(*g*_*c*_)*t*(*g*_0_−*g*_*c*_,…,*g*_*P*−1_−*g*_*c*_) where *t*(*g*_*c*_) defines the intensity distribution of I(*x*, *y*) that contains no useful information in order to describe local texture pattern. Hence only the joint distributions of differences are used to model the local texture: *T*≈*t*(*g*_0_−*g*_*c*_,…,*g*_*P*−1_−*g*_*c*_). By considering the signs of the differences, invariance with respect to gray-scale shifts is achieved: $T\approx t\left(s\left(g_{0}-g_{c}\right),...,\;s\left(gp-1-g_{c}\right)\right)\;s\;(x)=\;\{{{}_{0,\;}^{1,}}^{x\;\geq \;0}$

This is transformed into a unique P-bit pattern LBP code using Eq. 1. Thus the LBP code is computed by summing the threshold differences weighted by powers of 2. This gives a label for the center pixel that describes the local texture information.

(1) $$L\,B\,P_{P,R}=\sum_{p=0}^{P-1}s\,\left(g_{p}-g_{c}\right)\,2^{p}$$

Eq. 1 characterizes the spatial structure of the local image texture. The LBP histogram that describes the distribution of LBP values of a particular image patch is treated as its texture in classification problem.

It has been shown that the fundamental properties of texture can be captured by using only certain LBPs. These patterns are termed as “uniform patterns.” In our study also we observed that most of the LBPs present in a specific region of CC were uniform in nature. Uniformity “U” is measured by number bitwise transitions in the pattern. Patterns that have a “U” value of at most two are designated as “uniform.” The uniform LBP histogram is formed by considering separate output label for each uniform pattern whereas all the non-uniform patterns are allocated to a single label.

#### LBP Texture Feature Extraction

For the 2D TA approach, the uniform LBP values are calculated from each selected ROI for all subjects in the data set using MATLAB R2018a. As the sizes of ROIs are very small, we choose only one combination of neighborhood pixels: *P* = 8 with radius *R* = 1. Our previous study on TA of PSP has proved LBP energy and entropy as the best features as they contribute highest statistically significant textural difference in entire CC than all other extracted first order LBP histogram features like skewness, kurtosis, mean, variance, and mean absolute deviation (MAD) (Bhattacharya et al., 2019). Based on this observation, in the current study, we have computed gray level LBP histogram features Entropy and Energy in all ROIs which are considered as a descriptor of the distributions of the LBP texture values.

***(A) Entropy:*** Entropy is a measure of randomness of gray level distribution. Images with larger number of gray levels have larger Entropy. It is calculated using Eq. 2 as:

(2) $$H=-\sum_{i=0}^{n-1}f_{i}\,l\,o\,g_{2}\,\left(f_{i}\right);f_{i}\neq 0$$

where i denote the number of gray levels in each ROI and *f*_*i*_ is the frequency of occurrence of the sample values of LBP histogram.

***(B) Energy:*** Energy measures the intensity variation in an image patch and is calculated using Eq. 3 as:

(3) $$E=\sum_{i=0}^{n-1}{\left(x_{i}\right)}^{2}\,f_{i}$$

where the sample values of LBP histogram is denoted by *x*_*i*_.

#### Support Vector Machine Classification

The computed LBP texture features (on average 3,500 features for Energy and 3,500 for Entropy) are fed for training to off-the-shelf two-class classifier for the following scenarios: (a) PD-NC Vs. HC, (b) MSA Vs. HC, and (c) PSP Vs. HC. The purpose of this two class classification of each disease groups and HC group was to test the study hypothesis that there is anatomically coherent tissue loss in CC as a result of Parkinsonism. Sections “CC Sub-Region Ranking Based on Classification Performance” and “Scatter Index: Quantification of Dispersion in AI” of this paper describe how the result of this classification is utilized to form a new statistical framework that could be useful for differential diagnosis. Classification is performed for each ROI separately in order to detect the region of texture alteration within CC. In this regard we have used support vector machine (SVM), which is considered as a subfield of artificial intelligence.

**FIGURE 3:**
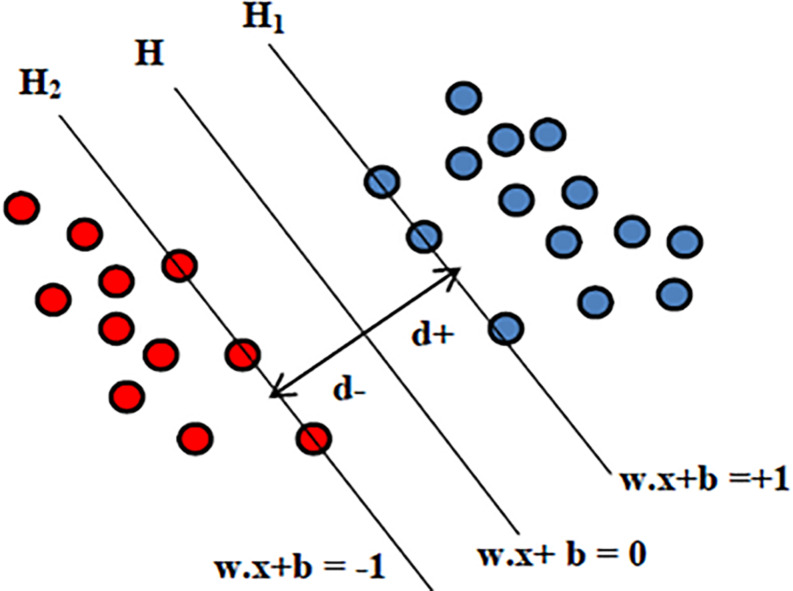
Illustration of SVM hyperplane.

Support vector machine is the supervised machine learning model where a model is learnt from known classes (labeled training data) and able to perform discriminative classification tasks by constructing hyperplanes (decision surface) in a multidimensional space that separates cases of different class labels. Support vectors are the data points that lie closest to the hyperplane and hence most difficult to classify. SVMs maximize the margin around the separating hyperplane. So, the hyperplane that has the largest distance to the nearest training-data point of any class will achieve good separation, as larger margin yields low generalization error of the classifier. The illustration of SVM hyperplane is shown in Figure 3.

Given the input features *x*_*i*_ = {*x*_1_,*x*_2_,…,*x*_*n*_}, the output for SVM will be the weights *w*_*i*_, one for each feature whose linear combination predicts decision boundary. The hyperplane H is defined using the straight line equation *wT*^*x*^ + *b* = 0, where w is the weight vector, *x* is the input vector and b is the bias. This will allow computing two planes H_1_ and H_2_ such that:

$$H_{1}:w^{T}\,x+b\geq 0,\text{if}\,d_{i}=+1$$

$$H_{2}:w^{T}\,x+b≺0,\text{if}\,d_{i}=-1$$

where d is the margin of separation between the hyperplane and the closest data point for a given weight vector w and bias b. The optimal hyperplane is the particular hyperplane for which the margin of separation d is maximized. The distance from a data point that lies on H_1_ or H_2_ to the hyperplane H is computed as |*w*^*T*^*x* + *b*|/|*w*|. Hence in order to maximize the margin of separation “d” one needs to minimize |*w*| so that there are no data points between H_1_ and H_2_. The problem of finding the optimal hyperplane can be solved by optimization techniques. In addition to performing linear classification, SVMs can also efficiently execute a non-linear classification problem where cluster analysis might not be a good choice. Non-linear SVM classification is performed using the kernel function that indirectly maps the data points into high-dimensional feature spaces. In this study the well-known sequential minimal optimization (SMO) algorithm with a polynomial kernel is used. As the principle of SVM lies in maximizing the margin to separate classes, the trained SVM model generalizes best on unseen data compared to other classifiers, making it a powerful tool at recognizing subtle patterns in complex biological datasets where feature patterns may represent the disease subtypes. Moreover SVM-based classification has the flexibility in choice of diverse kernels so as to optimize the performance even when target classes are overlapping. In problems with limited data instances, where deep neural networks cannot be utilized, SVM would be a best choice. To address the possibility of overfitting due to smaller number of subjects, our study considers leave-one-out cross validation where at each iteration the SVM model is trained on all subjects expect one subject and test error or prediction is computed for this held out subject.

### CC Sub-Region Ranking Based on Classification Performance

#### CC Sub-Division

Characterization of CC sub-regions is performed in order to check the degree of mid-sagittal callosal tissue alteration. Hence five well-defined anatomical sub-regions are generated by well-known Witelson and Hofer-Frahm CC vertical sub-division scheme (Hofer and Frahm, 2006) for each subject. In this study these five sub-regions denoted as R1, R2, R3, R4, and R5. Witelson’s CC sub-division scheme is based on primate data that divides the entire CC into five sub-regions which are as follows:

(a) Anterior third (R1): It consists of first one-third of entire CC. Rostrum, genu, and rostral body are assigned to pre-frontal, pre motor, and supplementary motor cortical areas.
(b) Anterior mid-body (R2): It consists of one-sixth of entire CC. Fibers originating in motor cortex are assumed to cross CC through anterior mid-body.
(c) Posterior mid-body (R3): It consists of one-sixth of entire CC. Somaesthetic and posterior parietal fiber bundles cross CC through this area.
(d) Posterior third (R4): It is two-fifteenth of entire CC that consist of posterior parietal and superior temporal fiber projection.
(e) Posterior one-fifth (R5): It is one-fifth of entire CC that consists of occipital and inferior temporal fiber projection.

However Witelson’s classification schemes could not able to reflect CC texture at the cellular level. Hence most of the current studies on CC prefer Hofer-Frahm scheme where parcelation of CC is done with respect to the outcome of the DTI fiber tractography that address cortical interconnectivity information of CC. According to Hofer-Frahm scheme the five vertical sub-regions of are as follows:

(a) Region1 (R1): It consists of the first one-sixth of entire CC and comprises fiber projecting into prefrontal area of cortex.
(b) Region-2 (R2): It consists of one-third of entire CC and comprises fibers that projects to premotor and supplementary motor areas.
(c) Region-3 (R3): In consists of one-sixth of entire CC and contains fibers projecting into the primary motor cortex. Hence in this particular region remarkable difference compared to Witelson’s classification was recognized which postulates that primary motor fiber cross CC in anterior half.
(d) Region-4 (R4): It is one-twelfth of entire CC. Primary sensory fibers cross CC through this region.
(e) Region-5 (R5): It is defined as posterior one-fourth. Parietal, temporal, occipital fiber cross CC through this region.

**FIGURE 4:**
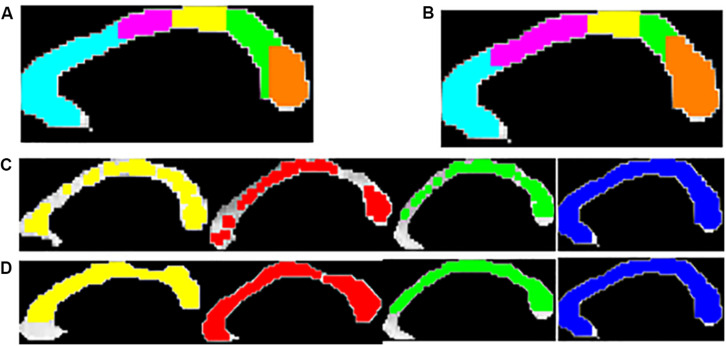
Five anatomical sub-regions of CC using Witelson’s **(A)** and Hofer-Frahm **(B)** scheme is shown. Visualization of the local texture alteration using Energy **(C)** and Entropy **(D)** is shown for one representative subject of PD-NC (yellow), MSA (red), PSP (green) and HC (blue). The colored region showed the ROIs that were correctly classified based on LBP texture features.

The five distinct CC sub-divisions according to Witelson’s and Hofer scheme are shown in Figure 4.

#### Correct Classification Ratio

We utilize a measure “correct classification ratio” (CCR) for each of the classes based on SVM classification performance on the individual extracted ROIs as described in section “Support Vector Machine classification.” The CCR values in each disease group quantify the extent of callosal tissue alteration that occurred due to Parkinsonism. This enables one to identify the CC sub-regions that are most affected by texture alteration in Parkinsonian disorders. CCR is defined as the ratio of number of correctly classified ROIs to the total number of ROIs for a specific sub-region. Hence for a particular subject, in a specific class, CCR depicts the fraction of ROIs that are correctly classified. Using both Witleson and Hofer schemes, in each sub-region, CCR values are calculated for each subject.

#### Detection of Key Callosal Sub-Regions With Highest Tissue Alteration

CCR values are used in order to identify best distinguishing callosal sub-region that corresponds to highest texture alteration. The following steps are performed separately for each of the classes to detect key callosal sub-regions with highest texture alteration:

Step 1: For each subject in the given class, the CCR value for every sub-region is sorted in descending order. This will result in an array of five components for five callosal sub-regions, where the first element contain CC sub-region index having highest CCR value and the last element contain CC sub-region index having least CCR. Hence we obtain a sorted array for each subject.

Step 2: These subject-specific sorted arrays are stacked together to form a matrix, of size 20 × 5, called the “CCR-Rank Matrix” (CCR-RM). Thus CC sub-region index of first column of the CCR-RM will contain the best-performing sub-region having highest texture alteration. We name this column as “*Array of Importance (AI)*,” reflecting the CC sub-regions with highest texture alteration.

Hence we defined the key callosal sub-regions as the sub-regions with highest number of occurrences in AI. The CCR values in AI are utilized to differentiate the disease groups.

### Scatter Index: Quantification of Dispersion in AI

The AI as defined in section “Detection of Key Callosal Sub-Regions With Highest Tissue Alteration” is utilized to capture the variation in distribution of CC sub-regions, across all subjects, for each disease group. For a given class, the frequency of occurrence of CC sub-regions across all subjects is used to measure the Frequency Standard Deviation (FSD) and Frequency Coefficient of Variation (FCV) using Eq. 4. If a certain CC sub-region index (say, sub-region *–* “*x*”) performs best classification across all subjects, in a given disorder; it would mean that the texture alteration is highly localized within that sub-region for the considered disorder. Conversely, if the sub-regions that performed best in classification vary among subjects it would mean that the texture alteration is spread over several sub-regions for that class. This leads to the notion of “Scatter index (SI),” which we propose to measure the degree of spread in texture alteration for a disorder. SI is computed using Eq. 5. Significance of SI lies in predicting the consistency in texture alteration in a particular sub-region for a particular disorder. Table 2 illustrates difference between the SI values of the two extreme cases: when (a) all sub-regions in AI appear equal number of times or (b) only one sub-region appears, for all subjects in AI. Considering these two ideal cases, it has to be noted from Table 2 that the ideal values of SI ranges from 0.135 to 1 if frequencies are distributed in five sub-regions, as is the case with the CC. Hence for our study we can infer that in a specific class if SI is close to 0.135, the tissue alteration is more localized for the given disorder. Similarly in a specific class if SI is close to 1, the tissue alteration is more scattered for that disorder.

**TABLE 2:** Ideal values of Frequency Standard Deviation (FSD), Frequency Coefficient of Variation (FCV), and Scatter Index (SI) values in two extreme cases when considering five sub-regions.

**Frequency of occurrence**	**1**	**2**	**3**	**4**	**5**	**FSD**	**FCV**	**SI**	**Interpretation**
Uniform occurrence	0.2	0.2	0.2	0.2	0.2	0	0	1	Regions with texture alteration are maximally scatter
Single occurrence	0	0	0	1	0	0.4	2	0.135	Regions with texture alterations are maximally localized

(4) $$F\,S\,D=\sqrt{\frac{\sum_{k=1}^{N}{\left(f_{k}-\bar{f}\right)}^{2}}{N}}\,\text{where}\,\bar{f}=\frac{\sum_{k=1}^{N}f_{k}}{N}$$

(5) $$S\,I=e^{-F\,C\,V}\,\text{where}\,F\,C\,V=F\,S\,D/\bar{f}$$

Here, “N” is the total number of CC sub-region (*N* = 5) and *f*_*k*_ is the frequency of occurrence of CC sub-regions.

Thus the percentage deviation from minimum value of SI in ideal case (here, *SI*_*IdealMin*_ = 0.135) will reflect how localized the texture alteration is across all CC-sub-regions.

(6) $$\%SI=\left(SI_{C\,a\,l\,c\,u\,l\,a\,t\,e\,d}-SI_{I\,d\,e\,a\,l\,M\,i\,n}\right)\times 100$$

Hence the smaller value of %SI will indicate more localized texture alteration within a particular sub-region.

## Results

The entire code was written in MATLAB R2018a and run on a machine with Intel^®^ Core^TM^ i3 4005U CPU 1.70 GHz processor with 4.0GB RAM. Figures 4C,D showed the visualization of ROIs which are correctly classified based on SVM classification using LBP-based features, Entropy and Energy. The mean CCR value in each callosal sub-region according to well-known Witelson’s and Hofer-Frahm’s scheme is calculated for extracted features and tabulated in Table 3. High CCR values are obtained using Entropy throughout CC compared to that for Energy. Using Energy the highest CCR values are obtained in sub-region R3 and R4 of Hofer’s CC sub-division scheme and in sub-region R2 to R4 of Witelson’s CC sub-division scheme.

**TABLE 3:** The mean Correct Classification Ratio (CCR) values, using the two-class classification accuracies obtained on ROIs separately in each of the five CC sub-regions.

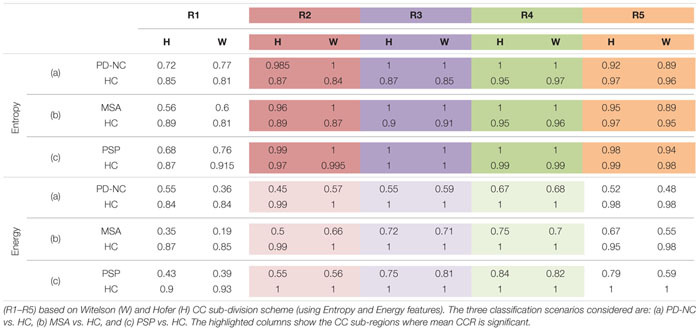

### Highest Tissue Alteration Is More Localized in Mid Callosal Region of PSP and MSA Compared to PD-NC

As discussed earlier in section “CC Sub-Region Ranking Based on Classification Performance,” AI of Parkinsonian disease groups computed from CCRM are examined to predict the sub-regions with highest texture alteration as compared to HC. Frequency of occurrence of CC sub-regions in AI is utilized (Table 4) to investigate the pattern of sub-regions distribution across all subjects in a particular disease group corresponding to highest texture alteration.

**TABLE 4:** Fraction of total CC sub-region occurrences (*f*_1_–*f*_5_) computed from Array of Interest (AI) using Witelson (W) and Hofer (H) scheme.

**Region**		**R1**	**R2**	**R3**	**R4**	**R5**

**Fraction of total occurrences**		***f*_1_**	***f*_2_**	***f*_3_**	***f*_4_**	***f*_5_**
PD-NC	H	0.1	0.2	0.3	0.3	0.1
	W	0.05	0.30	0.35	0.2	0.1
MSA	H	0	0.25	0.4	0.35	0
	W	0	0.25	0.5	0.25	0
PSP	H	0	0.05	0.45	0.50	0
	W	0	0.1	0.35	0.55	0

**FIGURE 5:**
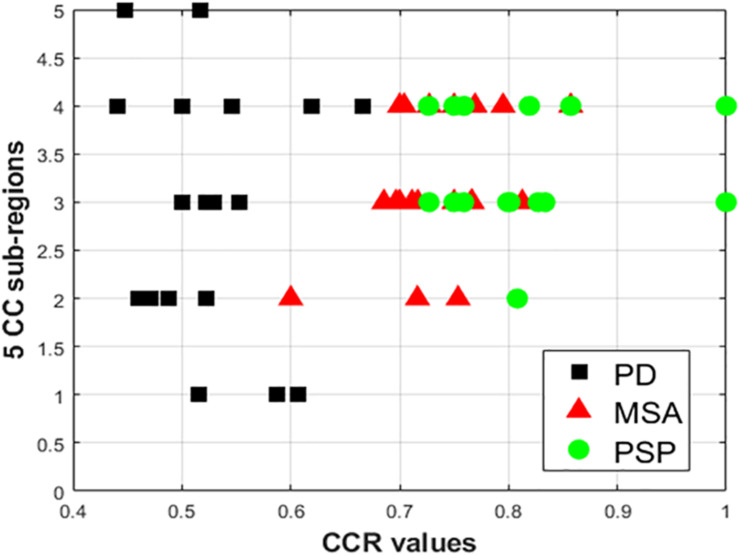
Plot of frequency of occurrence of CC sub-regions using energy feature, based on entries in AI (*X* axis represents CCR values; *Y*-axis represents the five distinct CC sub-regions). The vertical spread for PD-NC (black) indicates greater spread of maximum texture altered sub-regions in AI for all PD subjects, compared to MSA(red) and PSP (green) where for all subjects maximum texture alteration was mostly concentrated at mid-body (R3 and R4) for all respective subjects.

Comparing all disease groups, it is observed that most of the occurrences of CC sub-region in AI of PSP group are localized into two regions: R3 and R4. The same is observed in MSA and PD-NC. Using LBP Energy feature the frequency distribution of CC sub-regions according to Hofer scheme for each disease group is plotted in Figure 5. More concentration of subjects in R3 and R4 in Figure 5 clearly depicts mid callosal regions as the key regions with highest texture alteration for all disease groups. Using Hofer scheme, the percentage of subjects in AI corresponds to key sub-regions (R3 + R4) are as follows: (a) PD-NC: 60% (12/20^1^), (b) MSA: 75%, (15/20), and (c) PSP: 95% (19/20). Similar result is observed using Witelson scheme. Using this scheme, 65% PD-NC (13/20) and 75% of MSA (15/20) subjects corresponds to key sub-region R2 and R3, whereas 90% of PSP (18/20) subjects correspond to key sub-region R3 and R4. Using Entropy it was seen that almost all subjects of each class gives maximum tissue alteration from R2 to R5.

The observation made from Figure 5 in turn illustrates how scattered or localized the tissue alteration is across all subjects in a specific class. It is observed that frequency of occurrence of CC sub-regions across all subjects are more scattered from R1 to R5 in case of PD-NC (shown in black in Figure 5) compared to MSA (shown in red in Figure 5) and PSP (shown in green in Figure 5) where frequency of occurrence of CC sub-regions across all subjects are concentrated mostly in mid callosal regions. This is further verified quantitatively using SI to check how localized the texture alteration is in AI across all subjects for a specific class. The computed FSD, FCV, and SI values using Energy for each disease groups are shown in Table 5. Using Entropy the SI is found to be 0.13 (equals to ideal value of SI) as maximum texture alteration for all subjects using entropy was found in R3 and R4 using Hofer scheme and in each R2, R3, and R4 using Witelson scheme.

**TABLE 5:** Scatter Index (SI) Values (using Energy) for Parkinsonian disorders obtained from AI: using Hofer (H) and Witelson (W) schemes.

**Disorders**		**FSD**	**FCV**	**SI**	**% SI**
PD-NC	H	0.10	0.5	0.6	47
	W	0.12	0.6	0.54	41
MSA	H	0.19	0.95	0.38	25.1
	W	0.2	1	0.36	22.5
PSP	H	0.25	1.25	0.28	15.1
	W	0.24	1.2	0.3	16.6

### CCR Values of AI Obtained Using LBP Energy Feature Address Differential Diagnosis

**FIGURE 6:**
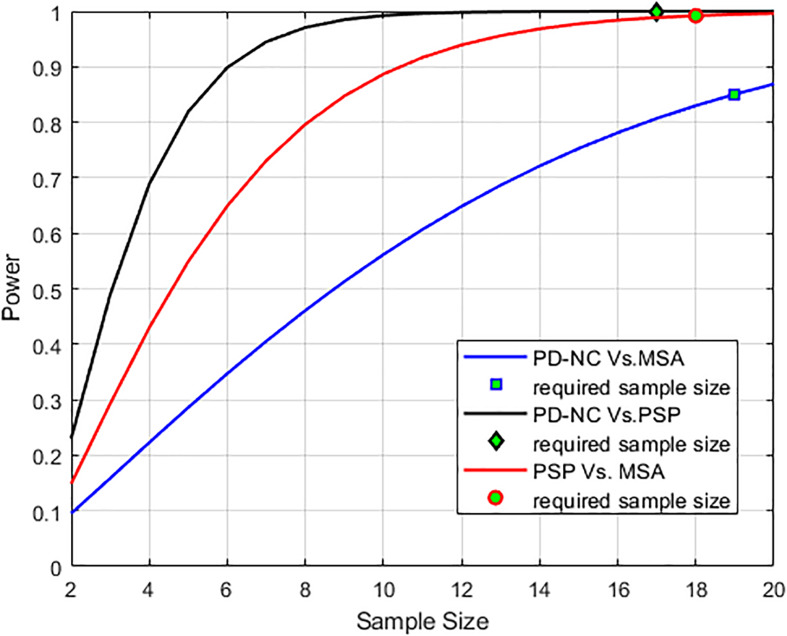
Power analysis curve is plotted that showed high statistical power of the analysis. The minimum number of sample size that is required (*n* = 17 to classify PD-NC vs. PSP, *n* = 18 to classify PSP vs. MSA and *n* = 19 to classify PD-NC vs. MSA) to obtain the desired power is also shown.

**FIGURE 7:**
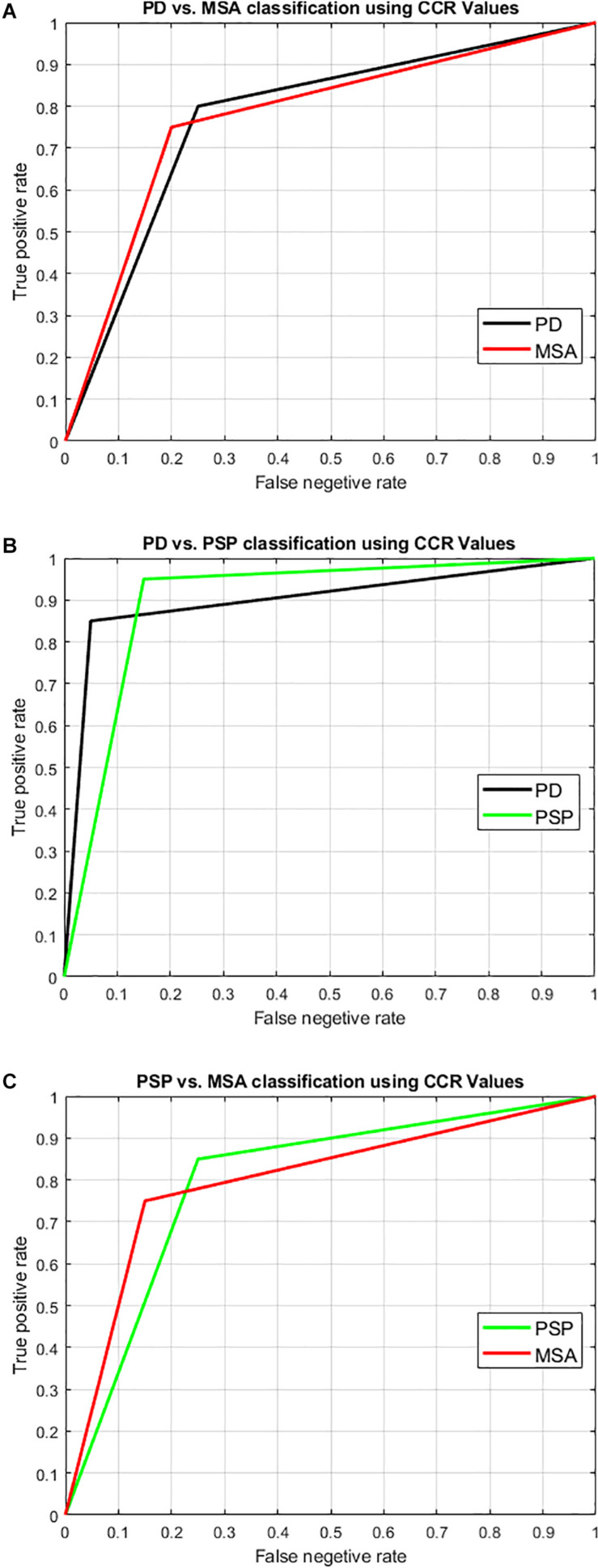
Illustration of result of differential diagnosis using Receiver operator characteristics (ROC) curve. SVM with leave-one-out two-class classification was performed to classify the disease groups to check the potential of our technique to perform differential diagnosis. The two-class classification scenarios were: **(A)** PD-NC Vs. MSA, **(B)** PD-NC Vs. PSP, and **(C)** PSP Vs. MSA. The ROC curve pertaining to the classification into each group is shown here with the obtained result as tabulated in Table 6. The area under the curve (AUC) is obtained as 0.825, 0.90, and 0.8 relating to both groups in each of the classification scenarios respectively. Comparing all disease groups, the highest accuracy with AUC over 0.9 was obtained when classifying typical PD (PD-NC) with respect to PSP.

Using LBP energy texture feature, the mean CCR in AI for PD-NC, MSA, and PSP is obtained as 0.54 ± 0.07, 0.73 ± 0.05, and 0.86 ± 0.11 respectively which represent the amount of correctly classified ROIs corresponding to highest texture alteration. This diversity in CCR values in AI among disease is exploited to distinguish the disease groups, leading to differential diagnosis. Student paired t-test is executed, confirming that the difference in CCR values is statistically significant between the disease groups (*p* = 1.5 × 10^–7^ for PD-NC and PSP, *p* = 1.78 × 10^–5^ for MSA and PSP, *p* = 0.0015 for PD-NC and MSA). Power analysis is performed using MATLAB to compute power that checks the probability of rejecting the null hypothesis, when the null hypothesis is false. Given the sample size, the power analysis results statistical power of 0.8690 between PD-NC and MSA, 1 between PD-NC and PSP, and 0.9961 between MSA and PSP. The high statistical power of the study indicates lower probability of making type-II error. The power curve is plotted in Figure 6 that also showed the minimum size of data sample that is required to provide the obtained power. The energy CCR values in AI are fed to SVM for two class classification of (a) PD-NC Vs. MSA, (b) PD-NC Vs. PSP, and (c) MSA Vs. PSP using polynomial kernel function. The performance measures of SVM for disease classification are reported in Table 6. The receiver operator characteristic (ROC) curves (Figure 7) plot the true-positive rate of classification (sensitivity) against the false-positive rate (specificity) for three disease classification scenario. The maximum accuracy of 90% accuracy is obtained to classify PD-NC from its atypical variant of PSP with area under the curve (AUC) of 90% relating to both groups. The obtained classification accuracy is 80 and 82.5% in classifying between MSA and PSP, PD-NC, and MSA, respectively.

**TABLE 6:** Performance measures of Support Vector Machine (SVM) for differential diagnosis using Correct Classification Ratio (CCR) values that reflects the extent of callosal tissue alteration in Parkinsonian disorders.

	**Sensitivity^a^**	**Specificity^b^**	**Precision^c^**	**Recall^d^**	***F*-score^e^**	**Accuracy (%)**
PD-NC	0.8	0.85	0.84	0.8	0.82	82.5
MSA	0.85	0.8	0.81	0.85	0.83	
PD-NC	0.85	0.95	0.94	0.85	0.89	90
PSP	0.95	0.85	0.86	0.95	0.9	
MSA	0.85	0.75	0.77	0.85	0.81	80
PSP	0.75	0.85	0.84	0.75	0.79	

^*a*^Sensitivity = tp/(tp + fn), ^*b*^Specificity = tn/(tn + fp), ^*c*^Precision = tp/(tp + fp), ^*d*^Recall = tp/(tp + fn), ^*e*^*F*-score, a measure of classification performance, = 2*precision*recall/precision + recall, where tp = true positive, fp = false positive, tn = true negative, and fn = false negative.

## Discussion

Texture analysis of medical images is a growing research area since the medical image texture is rich in diagnostic information that can be exploited. There are number of literatures that report the application of TA in the field of medical image analysis (Freeborough and Fox, 1998; Kaeriyama et al., 2002; Kodama et al., 2009; Harrison, 2011; Sivapriya et al., 2011; Oppedal et al., 2015). TA of MR images is a quantitative tool that enables characterization of different tissue types by analyzing its textures. In this regard the statistical approaches are widely used in medical images that utilize the spatial distribution of gray values to derive a set of statistics from the distributions of the local features that are defined by the combination of intensities at specific position relative to each pixel in image. The use of gray level co-occurrence matrices (GLCM) and run-length matrices (Haralick, 1973; Kaeriyama et al., 2002) are the most popular methods to study different neurological disorders. Statistically significant GLCM texture differences of CC was found in subjects with mild Alzheimer disease, amnestic mild cognitive impairment (Sivapriya et al., 2011), and also with mild traumatic brain injury (Holli et al., 2010). Classification of dementia in AD using TA of brain MRI with wavelets and GLCM results in 90–97% accuracy (Sivapriya et al., 2011). GLCM and run length matrix based texture parameters to differentiate among patients with AD, those with Lewy bodies, and HC subjects showed an accuracy of 91.7, 70.0, and 88.0%, respectively (Kodama et al., 2009). Despite of wide applicability the high dimensionality of GLCM and run-length-based texture parameter is one major limitation as it increases computational complexity. In such situation, LBP TA is an alternate way which is computationally simple yet has been found very efficient in the studies of medical texture classification (Oppedal et al., 2015).

In this study, we have demonstrated the ability of MRI-based LBP TA to characterize the CC sub-regions in different Parkinsonian disorders, and the utility of this method lies in the possible diagnosis of atypical Parkinsonian disorders. All possible ROI which are well within CC are extracted, followed by computing LBP texture features like Energy and Entropy. For each ROI, SVM classification using these texture parameters is performed for each disease group with respect to HC in order to predict the texture alteration caused due to neurodegeneration. ROIs that yield the correct classification are visualized and quantified further for characterizing CC sub-regions based on highest texture alteration. For this, AI is computed from CCRM that predicts the sub-region where changes in callosal tissue is maximum. AI detects the callosal mid-body as the key region with highest texture alteration.

The proposed index SI computed from AI is intended to measure the degree of spread of texture alteration in CC. Percentage deviation in SI identifies how localized the texture alteration is within a particular sub-region. Thus less the value of percentage SI, more localized the tissue alteration is within CC. Although using Witelson and Hofer schemes, Energy texture features showed significant tissue alteration at mid-body of CC, for Entropy it was significant enough from mid-anterior to extreme posterior of CC with the value of mean CCR in the range of 0.95 to 1. Using Energy the maximum percentage of SI is obtained for PD-NC (47% according to Hofer scheme) indicating that occurrence of CC sub-regions in AI and so the texture alteration is scattered across all subjects and could be the cause of attaining minimum mean CCR score (0.55 ± 0.07) at callosal mid-body. However, in case of PSP and MSA, occurrences of CC sub-regions in AI with highest tissue alteration are more localized at mid-body region (R3 and R4) with percentage of SI at 25.1% for MSA and 15.1% for PSP according to Hofer scheme.

In comparing the three disease groups, it is found that the texture alteration becomes significant with disease severity. This is evident from the mean CCR values of AI that indicate the extent of callosal tissue loss due to Parkinsonism. The varied range of mean CCR of AI for the disease groups makes our study a potential tool, leading to differential diagnosis. Highest accuracy of 90% is obtained while distinguishing PSP and PD-NC. The accuracy reduced to 82.5% when differentiating MSA from PD-NC. This could be attributed to the fact that microstructural callosal tissue alteration is more pronounced in PSP compared to MSA and PD-NC. This is further verified from the callosal centile thickness profile which is plotted in Figure 2 that clearly depicts reduction in callosal thickness is more prominent in PSP than MSA when they are compared with the thickness profile of PD-NC. The classification of PSP and MSA results accuracy of 80%.

The CC is the main white-matter fiber bundle and has several critical connections with cortical regions. The current knowledge of callosal involvement in neurodegenerative disorders suggest that the changes in motor cortical activation are more pronounced in case of Parkinsonian disorders (Defebvre et al., 1999; Halliday et al., 2005; Jennifer, 2011; Worker et al., 2014; Fichera et al., 2016). Axonal degeneration in the CC specifically in the axons in the mid body of the CC interconnect areas of the motor cortex that is responsible for preparation and sensory guidance of movement and abnormalities in this segment are proposed to be secondary to damage in cortical neurons, i.e., due to Wallerian degeneration (Hellier, 2014). The findings of this study demonstrate the presence abnormalities in the CC of PD and atypical Parkinsonian disorders, and the possible utility in automated diagnosis.

The novelty of the paper lies in deriving SI as a measure in order to detect the most-distinguishing CC sub-regions with highest tissue alteration based on LBP texture. The proposed method showed callosal texture alteration is more localized at callosal mid-body for atypical variants of PD. Using SI, the quantification of changes in callosal tissue at its mid-body could be used as a possible biomarker tool in computer aided diagnosis of Parkinsonian disorders. The study is limited by data availability and needs to be conducted on a much larger dataset for generalization. Nevertheless the inference from the current study offers a basis for practical clinical applications that could address differential diagnosis. However it is to be noted that CC is not a primary site of pathology in Parkinsonian syndromes as it is involved at advanced stages of disease. Therefore, the proposed study highlights the potential of the technique but future studies need adaptation to anatomical structures that are subject to degeneration at earlier stages of Parkinsonian syndromes.

## Conclusion

In this study we estimated the proficiency of LBP TA for characterizing CC sub-region based on its textural alteration that occurred in patients with Parkinsonian disorders. We hypothesized that these neurodegenerative diseases may cause loss in tissue structure of CC which is not perceptible visually. A new measure, “SI index,” is proposed to predict the CC sub-regions that are best classified across all subjects for a particular class using LBP TA. Our proposed methodology showed mid-body of CC as the key region with significant texture deviations in case of Parkinsonian disorder compared to HC. Hence, the study of LBP with mid-body CC TA showed its potential to become a new additional tool, to aid in the detection of CC texture alterations in Parkinsonian disorders for the diagnosis and understanding of this pathology.

## Data Availability Statement

The raw data supporting the conclusions of this article will be made available by the authors, without undue reservation, to any qualified researcher.

## Ethics Statement

We confirm that we have read the Journal’s position on issues involved in ethical publication and affirm that this report is consistent with those guidelines.

## Author Contributions

DB has been resposible for designing and drafting the work, analysis, and interpretation of data for the work. NS has contributed towards conception, analysis, interpretation of data for the work. The research work was performed by DB under the guidance of NS. JS has provided his valuable conception, design of the work; acquisition and interpretation of data for the work. Material collection, data preparation, analysis, interpretation of data for the work has been performed by SM, PP, and SP. The final draft of the manuscript has been critically revised by DB, NS, JS, and SP for important intellectual content. All authors are in agreement to be accountable for all aspects of the work in ensuring that questions related to the accuracy and integrity of any part of the work are appropriately investigated and resolved. All authors have read and approved the final manuscript. All authors have contributed to the study conception and design.

## Conflict of Interest

The authors declare that the research was conducted in the absence of any commercial or financial relationships that could be construed as a potential conflict of interest.

## Footnotes

1 No. of subjects with highest CCR obtained in key sub-regions/total no. of subjects.

## References

[B1] Bhattacharya D. Vengalil S. Sinha N. Saini J. Pal P. Sandhya M. (2019). “Structural MRI based texture analysis of corpus callosum in classifying Progressive supranuclear palsy,” in *Proceedings of the IEEE Region 10 Conference TENCON 2019*, Kochi, 441–446. 10.1109/TENCON.2019.8929403

[B2] Bishop K. M. Wahlsten D. (1997). Sex differences in the human corpus callosum: myth or reality? *Neurosci. Biobehav. Rev.* 21 581–601. 10.1016/s0149-7634(96)00049-8 9353793

[B3] Defebvre L. J. Derambure P. Bourriez J. L. Cassim F. Guieu J. D. (1999). Motor programming is more affected in progressive supranuclear palsy than in Parkinson’s disease: a spatiotemporal study of event-related desynchronization. *Mov. Disord.* 14 634–641. 10.1002/1531-8257(199907)14:4<634::aid-mds1013>3.0.co;2-q 10435501

[B4] Fichera M. Houdayer E. Avantaggiato F. Chieffo R. Comi G. Volonté M. A. (2016). Motor cortical disinhibition is more pronounced in Progressive Supranuclear Palsy than in Parkinson’s disease: evidence from TMS. *Clin. Neurophysiol.* 127:e121.

[B5] Freeborough P. A. Fox N. C. (1998). MR image texture analysis applied to the diagnosis and tracking of Alzheimer’s disease. *IEEE Trans. Med. Imaging* 17 475–479. 10.1109/42.712137 9735911

[B6] Gilman S. Wenning G. K. Low P. A. Brooks D. J. Mathias C. J. Trojanowski J. Q. (2008). Second consensus statement on the diagnosis of multiple system atrophy. *Neurology* 71 670–676. 10.1212/01.wnl.0000324625.00404.15 18725592 PMC2676993

[B7] Halliday G. M. Macdonald V. Henderson J. M. (2005). A comparison of degeneration in motor thalamus and cortex between progressive supranuclear palsy and Parkinson’s disease. *Brain* 128 2272–2280. 10.1093/brain/awh596 16014651

[B8] Haralick R. M. (1973). Textural features for image classification. *IEEE Trans. Image Man Cybernet.* SMC-3, 610–621.

[B9] Harrison L. (2011). *Clinical Applicability of MRI Texture Analysis.* Ph.D. thesis, University of Tampere, Tampere.

[B10] Harwood D. Ojala T. Pietikäinen M. Kelman S. Davis S. (1993). *Texture classification by center-symmetric auto-correlation, using Kullback discrimination of distributions*. Technical report CAR-TR-678, College Park, Maryland: Computer Vision Laboratory, Center for Automation Research, University of Maryland.

[B11] Heim B. Krismer F. Seppi K. (2018). Structural imaging in atypical parkinsonism. *Int. Rev. Neurobiol.* 142 67–148. 10.1016/bs.irn.2018.08.010 30409261

[B12] Hellier J. L. (2014). *The Brain, the Nervous System and Their Diseases.* Santa Barbara, CA: ABC-CLIO.

[B13] Hofer S. Frahm J. (2006). Topography of the human CC revisited—Comprehensive fiber tractography using diffusion tensor magnetic resonance imaging. *Neuroimage* 32 989–994. 10.1016/j.neuroimage.2006.05.044 16854598

[B14] Holli K. K. Harrison L. Dastidar P. Wäljas M. Liimatainen S. Luukkaala T. (2010). Texture analysis of MR images of patients with mild traumatic brain injury. *BMC Med. Imaging* 10:8 10.1186/1471-2342-10-8 PMC3161385 20462439

[B15] Hughes A. J. Daniel S. E. Blankson S. Lees A. J. (1993). A clinicopathologic study of 100 cases of Parkinson’s disease. *Arch. Neurol.* 50 140–148. 10.1001/archneur.1993.00540020018011 8431132

[B16] Jennifer L. (2011). Clinical correlates of white matter tract degeneration in progressive supranuclear palsy. *Arch. Neurol.* 68 753–760. 10.1001/archneurol.2011.107 21670399 PMC3401587

[B17] Kaeriyama T. Kodama N. Shimada T. Fukumoto I. (2002). Application of run length matrix to magnetic resonance imaging diagnosis of Alzheimer-type dementia [in Japanese]. *Nippon Hoshasen. Gijutsu Gakkai Zasshi* 58 1502–1508. 10.6009/jjrt.kj00000921502 12568081

[B18] Kodama N. Kawase Y. Okamoto K. (2009). “Application of texture analysis to differentiation of dementia with Lewy bodies from Alzheimer’s disease on magnetic resonance images,” in *Proceedings of the World Congress on Medical Physics and Biomedical Engineering*, Vol. 14 Munich, 1444–1446.

[B19] Lenka A. Pasha S. A. Mangalore S. George L. Jhunjhunwala K. R. Bagepally B. S. (2017). Role of corpus callosum volumetry in differentiating the subtypes of progressive supranuclear palsy and early Parkinson’s disease. *Mov. Disord. Clin. Pract.* 4 552–558. 10.1002/mdc3.12473 30363434 PMC6174399

[B20] Litvan I. Hauw J. J. Bartko J. J. Lantos P. L. Daniel S. E. Horoupian D. S. (1996). Validity and reliability of the preliminary NINDS neuropathologic criteria for progressive supranuclear palsy and related disorders. *J Neuropathol Exp Neurol.* 55 97–105. 10.1097/00005072-199601000-00010 8558176

[B21] Luders E. Thompson P. M. Kurth F. (2018). “Morphometry of the corpus callosum,” in *Brain Morphometry: Non Clinical Applications*, eds Spalletta G. Gili T. Piras F. (Berlin: Springer).

[B22] Ojala T. (1996). *Multichannel Approaches to Texture Description with Feature Distributions.* Technical Report CAR-TR-846, Maryland: University of Maryland.

[B23] Ojala T. Valkealahti K. Oja E. Pietikäinen M. (2001). Texture discrimination with multidimensional distributions of signed gray-level differences. *Pattern Recogn.* 34 727–739.

[B24] Oppedal K. Eftestol T. Engan K. Beyer M. K. Aarsland D. (2015). Classifying dementia using local binary patterns from different regions in magnetic resonance images. *Int. J. Biomed. Imaging* 2015 572567. 10.1155/2015/572567 25873943 PMC4385607

[B25] Paviour D. C. Price S. L. Jahanshahi M. Lees A. J. Fox N. C. (2006). “Regional brain volumes distinguish PSP, MSA-P, and PD: MRI-based clinico-radiological correlations. *Mov. Disord.* 21 989–996. 10.1002/mds.20877 16602104

[B26] Rispoli V. Schreglmann S. R. Bhatia K. P. (2018). Neuroimaging advances in Parkinson’s disease. *Curr. Opin. Neurol.* 31 415–424. 10.1097/WCO.0000000000000584 29878908

[B27] Sivapriya T. R. Saravanan V. Ranjit J. Thangaiah P. (2011). Texture analysis of brain MRI and classification with BPN for the diagnosis of dementia. *Commun. Comput. Inform. Sci.* 204 553–563.

[B28] Timothy T. H. Kang X. Woods D. L. (2012). Automated measurement of the human corpus callosum using MRI. *Front. Neuroinform.* 6:25 10.3389/fninf.2012.00025 PMC3439830 22988433

[B29] Williams D. R. Litvan I. (2013). Parkinsonian syndromes. Continuum (Minneap Minn). *Mov. Disord.* 19 1189–1212. 10.1212/01.CON.0000436152.24038.e0 24092286 PMC4234134

[B30] Worker A. Blain C. Jarosz J. Chaudhuri K. R. Barker G. J. Williams S. C. (2014). Diffusion tensor imaging of Parkinson’s disease, multiple system atrophy and progressive supranuclear palsy: a tract-based spatial statistics study. *PLoS One* 9:e112638 10.1371/journal.pone.0112638 PMC4236070 25405990

